# *Cronobacter sakazakii* ST4 Strains and Neonatal Meningitis, United States

**DOI:** 10.3201/eid1901.120649

**Published:** 2013-01

**Authors:** Sumyya Hariri, Susan Joseph, Stephen J. Forsythe

**Affiliations:** Author affiliation: Nottingham Trent University School of Science and Technology, Nottingham, UK

**Keywords:** *Cronobacter sakazakii*, multilocus sequence typing, MLST, neonatal meningitis, clonal complex strains, bacteria, United States

**To the Editor**: To overcome various limitations of phenotyping and 16S rDNA sequence analysis of *Cronobacter* bacteria, we have established a comprehensive multilocus sequence typing (MLST) scheme as an open access database resource (www.pubMLST.org/cronobacter) (*1*). The scheme is based on 7 housekeeping genes (*atpD*, *fusA*, *glnS, gltB, gyrB*, *infB*, *ppsA*; 3,036 nt concatenated length) and has been used to study the diversity of the *Cronobacter* genus and new *Cronobacter* species (*2*–*4*). Previously, we compared the sequence type profile to severity of infection by compiling patient details, isolation site, and clinical signs and symptoms for strains isolated from around the world during 1953–2008 (*5*). This study revealed that most serious meningitis clinical cases caused by *Cronobacter* spp. in neonates during the previous 30 years in 6 countries were caused by a single sequence type (ST): *C. sakazakii* ST4. We were therefore interested in applying the MLST method to the *Cronobacter* strains associated with the highly publicized cases in the United States during December 2011 (*6*).

The Centers for Disease Control and Prevention (CDC) sent us the *Cronobacter* isolates they collected during 2011 for MLST analysis (Table). Ten specimens were clinical isolates from neonates or infants. These included 2 specimens (1577, 1579) associated with *Cronobacter* infections in Missouri and Illinois (*6*). Four specimens were from opened tins of powdered infant formula (PIF), and 1 was from PIF reconstitution water. DNA sequences for all specimens are available for download and independent analysis through the open access database.

**Table:** Multilocus sequence typing of *Cronobacter* isolates received by the CDC during 2011*

*Cronobacter* species	NTU strain ID no.	CDC patient ID no.	ST	Location	Isolation source	Comment
*C. sakazakii*	1579	2012-05-05	4	Missouri	CSF of <1 mo male, term infant; exposed to PIF	Patient died
	1566	2011-12-02	4	Ohio	CSF of 1 mo male infant; exposed to PIF	From twin of patient 2011-12-03
	1567	2011-12-03	4	Ohio	Feces of 1 mo male infant; exposed to PIF	From twin of patient 2011-12-02; asymptomatic
	1568	2011-12-04	4	Ohio	Opened PIF	Formula associated with 2011-12-02 and -03
	1570	2011-21-01	4	Minnesota	CSF of <1 mo male, term infant; exposed to PIF	Brain infarction
	1571	2011-21-03-01	4	Minnesota	Opened PIF	Formula associated with 2011-21-01
	1576	2193-02	4	Michigan	Tracheal secretion of <1 mo male, pre-term infant (30-wk EGA); not exposed to PIF	Symptoms were not caused by *Cronobacter* infection. Fortified breast milk fed only after culture was obtained
	1565	2011-12-01	107	Michigan	CSF of <1 mo male, term infant; exposed to PIF	Brain abscess; outcome unknown. Single locus variant of ST4
	1572	2011-21-03-02	108	Minnesota	Opened PIF	Single locus variant of ST4
	1577	2193-03	110	Illinois	CSF of 1 mo female, term infant; exposed to PIF	Triple locus variant of ST4
	1578	2193-08-01	111	Illinois	PIF reconstitution water	Bottled water associated with 2193-03 case
	1573	2011-18-05-02	8	Ohio	Opened PIF	Formula associated with 2011-18-01 and 2011-18-07
	1574	2011-18-01	8	Ohio	Feces of 4 mo female, term infant; exposed to PIF	Diarrheal symptoms
	1575	2011-18-07	8	Ohio	Feces of ≈5 mo female, term infant; exposed to PIF	Ongoing diarrhea; same patient as 2011-18-01
*C. malonaticus*	1569	2193-01	112	Wisconsin	Blood of <1 mo male pre-term infant (32 week EGA); exposed to PIF	Clinical meningitis; patient died

*NTU, Nottingham Trent University; ID, identification; CDC, Centers for Disease Control and Prevention; ST, sequence type; CSF, cerebrospinal fluid; PIF, powdered infant formula; EGA, estimated gestational age.

Most (14/15) specimens were *C. sakazakii*; 1 was *C. malonaticus*. This predominance of *C. sakazakii* isolates matches reports of cases and outbreak studies (*7*). The *C. sakazakii* isolates were in 6 of 55 STs defined for *C. sakazakii* (*4*). However, there was an uneven distribution according to clinical records: all 5 cerebrospinal fluid (CSF) isolates were either ST4 or within the ST4 complex (clonal group where strains are identical in 4 or more loci). This group included strains from cases during December in Illinois (specimen 1577) and in Lebanon, Missouri (specimen 1579).

Specimen 1577 (ST110), isolated from CSF, is a triple-loci variant of ST4, distinguished by 5/3036 nt: *atpD* (1/390nt), *gltB* (2/507nt), and *gyrB* (2/402nt). Specimen 1578 (ST111), isolated from the PIF reconstitution water associated with the case reported in Illinois, is distinguishable from ST4 in 4/7 loci: *fusA* (5/438nt), *glnS* (1/363), *infB* (4/441), and *ppsA* (19/495). The 2 Illinois strains, 1577 and 1578 (ST110 and ST111), differed from each other at all loci, in total, 35/3,036 nt difference.

Such sequence-based relationship analysis of isolates is not possible by using pulsed-field gel electrophoresis (PFGE). PFGE and MLST analyze the bacterial DNA content differently, and there are no *Xba*I sites (the endonuclease most commonly used with PFGE of Enterobacteriaceae) within the 7 MLST loci. *C. sakazakii* ST4 strains were also found in feces (specimen 1567), opened PIF (specimen 1571), and tracheal samples (specimen 1576) (Figure). In addition, 2 single-loci ST4 variants were found; CSF specimen 1565 differed from the ST4 profile in the *fusA* loci by 6/438 nt, and specimen1572 from an opened tin of PIF differed in the *fusA* loci by 5/438 nt. These 2 strains differ from each other minimally, by 1 nt of 3,036 (concatenated length) in the *fusA* loci position 378 (A:T).

**Figure:**
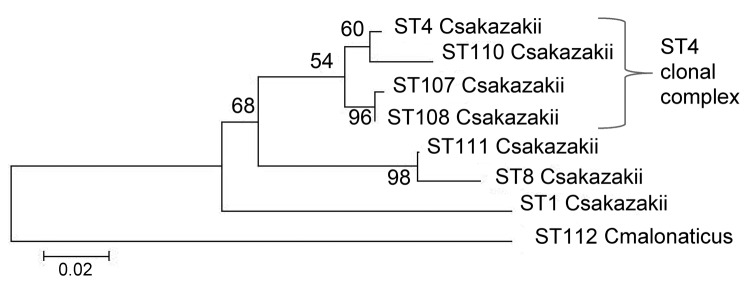
Maximum-likelihood tree based on the concatenated sequences (3,036 bp) of the 7 multilocus sequence type (ST) loci for *Cronobacter* isolates. The tree was drawn to scale by using MEGA5 (www.megasoftware.net), with 1,000 bootstrap replicates. Numbers on branches indicate percentage of bootstrap values. Scale bar indicates nucleotide substitutions per site.

Several non-ST4 *C. sakazakii* strains were received by CDC in 2011. *C. sakazakii* ST8 was isolated from an opened powdered infant formula tin (specimen 1573) and 2 associated fecal samples from an infant who had diarrhea (specimens 1574 and 1575). One blood isolate (specimen 1569) was *C. malonaticus* ST112, found in an infant <1 month of age with meningitis who did not survive the infection. This finding is highly noteworthy because it has been proposed that *C. malonaticus* predominates in adult infections (*5*), and no fatal meningitis cases have been attributed to this species.

This MLST analysis of 15 strains received by the CDC in 2011 reinforces the conclusion that CSF isolates are not evenly spread across the 7 *Cronobacter* species and are instead predominantly in the *C. sakazakii* ST4 clonal complex. Such infections in neonates are of high concern because of the risk for associated severe brain damage. As previously stated, whether this association is caused by greater neonatal exposure as a result of environmental factors or particular virulence capabilities remains uncertain (*5*).
